# Editorial: One Health approaches and modeling in parasitology in the climate change framework and possible supporting tools adopting GIS and remote sensing

**DOI:** 10.3389/fpara.2025.1560799

**Published:** 2025-03-19

**Authors:** Tommaso Orusa, Annalisa Viani, Silvio G. d’Alessio, Riccardo Orusa, Cyril Caminade

**Affiliations:** ^1^ INVA spa, Brissogne, Italy; ^2^ Istituto Zooprofilattico Sperimentale dell’Abruzzo e del Molise “G. Caporale”, Teramo, Italy; ^3^ Azienda Sanitaria Locale della Valle d’Aosta - AUSL Aosta - SC Sanità Animale, Quart, Italy; ^4^ Istituto Zooprofilattico Sperimentale del Piemonte, Liguria e Valle d’Aosta (IZS PLV) S.C Valle d’Aosta—CeRMAS (National Reference Center for Wildlife Diseases), Quart, Italy; ^5^ Earth System Physics, Abdus Salam International Centre for Theoretical Physics, Trieste, Italy

**Keywords:** One Health, modeling, parasitology, climate change, GIS, remote sensing

The interplay between climate change, parasitology, and the rise of emerging technologies presents a dynamic frontier for research aimed at safeguarding human, animal, and environmental health (Anyamba et al., 2019; Caminade, 2021; Orusa et al., 2023c). Within the One Health framework, an integrated approach that acknowledges the interconnection of these scientific fields (Ippoliti et al., 2019; Carella et al., 2022; Viani et al., 2023b) underscores the importance of utilizing geospatial technologies and predictive modeling in order to anticipate and address the risk posed by zoonotic parasitic diseases exacerbated by climate variability (Short et al., 2017; Orusa and Mondino, 2019; Orusa and Borgogno Mondino, 2021; Orusa et al., 2024a).

## 1 Introduction

The ongoing impacts of climate change are significantly affecting the epidemiology of parasitic diseases (Víchová et al., 2018), with rising temperatures, shifting precipitation, and extreme weather altering the habitats (Zhao et al., 2021; Viani et al., 2023c) and life cycles of vectors and pathogens (Adler et al., 2019; Afuye et al., 2021; Orusa et al., 2023b). Zoonotic diseases, which make up about 75% of emerging infectious diseases in humans, are on the rise (Viani et al., 2023a; Viani et al., 2025). Such increase is often linked to changes in wildlife behavior and habitat due to human activities. Technological advancements in Earth Observation (EO) (Orusa et al., 2024b), Geographic information systems (GIS), and remote sensing have enabled the creation of suitability maps (Anyamba et al., 2012; Orusa et al., 2022b; Viani et al., 2024b, a), disease tracking, and informed public health policies (Yu et al., 2017; Orusa et al., 2020, 2023a, a). Integrating these tools with high-resolution climate models and cloud-based platforms like Google Earth Engine (GEE) (Gorelick et al., 2017) allows for detailed spatial and temporal analyses, facilitating targeted interventions and risk assessments (Conte et al., 2015; Amdouni et al., 2022; Orusa et al., 2022a; Caminade et al., 2023; Arsevska et al., 2024). This Research Topic emphasizes the innovative use of geospatial tools and modeling techniques in Parasitology and One Health.

## 2 Insights from the contributing articles

The first contribution, “*Spatial prediction of the probability of liver fluke infection in water resource within sub-basin using an optimized geographically-weighted regression model*” (Pumhirunroj et al.) focuses on predicting the spatial distribution of liver fluke infections in sub-basin using an optimized geographically weighted regression (GWR) model. By analyzing infection data and environmental variables such as streams and the Normalized Difference Moisture Index (NDMI), the authors identified key predictors of infection fasciolosis risk. Model-3, which incorporated these evironmental variables, demonstrated superior accuracy, with an R² improvement from 0.754 to 0.800. The results emphasize the value of fine-scale spatial analyses in identifying high-risk areas and optimizing resource allocation for disease control.

The second review-article “*Amoebae: beyond pathogens - exploring their benefits and future potential*,” (Dinda et al.) explores the dual nature of amoebae as both harmful pathogens and beneficial contributors to ecosystems. Pathogenic species like *Entamoeba histolytica* and the brain-eating ameoba, *Naegleria fowleri*, pose significant health risks, while other species play crucial roles in nutrient cycling and in controlling plant pathogens. The authors highlight the potential of amoebae as model organisms in biomedical research and their promise for future biotechnological applications. This work broadens our understanding of amoebae’s ecological and scientific significance, offering a nuanced perspective about their role within the One Health framework.

Finally, the review “*Exploring extracellular vesicles in zoonotic helminth biology: implications for diagnosis, therapeutic, and delivery*” (Qadeer et al.) delves into the promising field of extracellular vesicles (EVs). By acting as mediators of intercellular communication, EVs from parasitic helminths offer novel avenues for early diagnosis and treatment. The potential for EV-based biomarkers to improve diagnostic specificity and therapeutic targeting is particularly significant in the context of zoonotic diseases. This article not only highlights cutting-edge research but also points to future applications in disease management and control.

## 3 Current and ongoing research perspectives

The studies presented in this Research Topic collectively underscore the potential of integrating GIS, remote sensing, and advanced modeling techniques into Parasitology. However, to fully take advantage of these technologies, future research should address several key areas:

### 3.1 High-resolution climate models

The development and application of climate models with ever-increasing spatial and temporal resolutions are critical for (i) detailed habitat analysis of vectors at local scales and (ii) anticipating future changes. These models can refine predictions of vector distributions, enabling more precise risk assessments and interventions.

### 3.2 Cloud computing and big data

Platforms like Google Earth Engine (GEE) are revolutionizing the processing and analysis of large-scale environmental datasets. By enabling access to global EO data and providing robust computational resources, GEE facilitates detailed analyses of vector habitats, land-use changes, and environmental suitability for disease transmission.

### 3.3 Dynamic risk mapping

Combining real-time EO data with predictive models enables the creation of dynamic risk maps. These maps can inform timely interventions, especially in data-sparse regions where climate change is accelerating shifts in vector habitats and disease patterns.

### 3.4 Cross-disciplinary collaboration

The One Health framework inherently requires collaboration across disciplines, including ecology, epidemiology, climatology, and public health. Strengthening these collaborations will enhance the development of holistic models that account for the complex interplay between environmental and socio-economic factors.

### 3.5 Local-scale analysis

While global and regional studies provide valuable insights, local-scale analyses are essential for effective implementation of interventions. Studies like the one about liver fluke infection model, demonstrate the utility of tailored approaches that consider specific environmental and socio-cultural contexts.

### 3.6 Open data platforms for wildlife georeferencing

Standardized, open-access platforms for georeferencing wildlife movements and habitats are critical for improving epidemiological studies. By systematically cataloging animal locations, researchers can better understand disease transmission dynamics and assess the impact of environmental changes on wildlife health.

### 3.7 Fixed monitoring areas for parasites

Establishing fixed monitoring sites for parasites such as ticks will enable the development of real-time exposure risk bulletins for domestic animals, livestock, and humans by integrating remote-sensed data. These bulletins could serve as early warning systems, empowering stakeholders to take preventive measures during high-risk periods.

The findings presented in this Research Topic have significant implications for policy and practice. The ability to generate high-resolution suitability maps and dynamic risk assessments can inform science-based policy decisions, guiding resource allocation and intervention strategies. Additionally, the integration of EO data and GIS modeling into national health systems can enhance surveillance and response capabilities, particularly in under-resourced and data-sparse regions (Chamaillé et al., 2010; Lambin et al., 2010; Cailly et al., 2012; Tran et al., 2016).

## 4 Conclusion

As climate change continues to reshape ecosystems and disease dynamics, the integration of advanced technologies and interdisciplinary approaches will be paramount in addressing emerging challenges. The studies featured in this Research Topic illustrate the potential of GIS, remote sensing, and modeling to enhance our understanding of parasitic diseases and inform effective interventions. By embracing these tools and fostering cross-sector collaborations, the One Health community can contribute to a more resilient and sustainable future.
